# Anesthetic Management for Heart Transplantation in Adults with Congenital Heart Disease

**DOI:** 10.31480/2330-4871/120

**Published:** 2020-03-27

**Authors:** Koichi Yuki

**Affiliations:** 1 Department of Anesthesiology, Critical Care and Pain Medicine, Boston Children’s Hospital, USA; 2 Department of Anaesthesia, Harvard Medical School, USA

## Abstract

As the outcome of patients with congenital heart disease (CHD) has improved, the number of adults with congenital heart disease (ACHD) outnumbered pediatric population with CHD. Heart failure is responsible for 40% of mortality among ACHD, and the number of heart transplantation for ACHD is gradually increasing. However, the early mortality rate of heart transplantation is significantly higher in ACHD than in non-ACHD. Understanding the unique characteristics of heart transplantation in ACHD is critical. In contrast to their early outcome their long-term survival is better in ACHD than in non-CHD patients, and they are likely to present to anesthesia care after heart transplantation for various reasons. Understanding specific issues in post-transplant anesthesia care is another important aspect.

## Introduction

Congenital heart disease (CHD) is the most common anomaly with an incidence of 4–8/1,000 live births [1]. With advances in surgical and medical management, the outcomes of CHD patients have significantly improved. Many people with CHD have lived into adulthood now so that adults with congenital heart diseases (ACHD) has outnumbered children with CHD [2]. In 2010 the number of CHD patients under 18 years of age was 1 million in the United States, whereas the number of ACHD was 1.4 million. More than one third of ACHD were greater than 45-years-old. In parallel, the prevalence of advanced heart failure among ACHD has increased. Now it is estimated that heart failure accounts for up to 40% of death in ACHD [3]. Persistent abnormalities in valvular and ventricular function together with arrhythmia burden and anatomical lesions including residual shunts are considered to put ACHD at very high risk of heart failure [4].

The heterogenous nature of ACHD complicates the universal application of typical heart failure therapies [5]. Accordingly, advanced heart failure in ACHD is usually managed by a multi-disciplinary team. Heart failure therapy includes medical therapy with heart failure medications, optimization of rhythm and other corrective procedures if indicated. Further advanced heart failure therapies include mechanical circulatory support (MCS) and heart transplantation. Based on the International Society of Heart and Lung Transplantation (ISHLT) 2015 registry, 3.3% of adult heart transplant recipients between 2009 and 2014 had CHD. The percentage of CHD among adult recipients was 2.7% from 2004 to 2008, and 2.0% from 1991 to 2003 [6], indicating the number of heart transplantation for ACHD increased over time. The most frequent CHD lesion was transposition of the great arteries (22%) and tricuspid atresia (8%) [4]. When classifying based on circulatory pattern, 40–50% of patients had single ventricle physiology with failing Fontan circulation [7,8].

Historically most ACHD have received low-priority on the heart transplant waiting list. As a result, ACHD tends to be on the waiting list for a much longer time than non-CHD patients, and these patients often seek medical care during this period. Thus, it is possible that these patients present to any local hospital for care at any stage of their heart failure. These transplantations have been performed widely not only in non-Adult Congenital Heart Association accredited centers but also in non-accredited centers [9]. These patients may also present to hospitals after heart transplantation. Thus, it is important for anesthesiologists to be familiar with this unique medical population. So far limited literature is available regarding anesthesia care of heart transplantation in ACHD, and this article serves to review the unique aspect of this population.

## Anesthetic Considerations for Heart Transplantation

ACHD patients who present for heart transplantation may be receiving a number of heart failure therapies including pharmacological intervention and synchronization therapy. Some may have MCS in place. Heart transplantation surgery consists of the pre-cardiopulmonary bypass (CPB) phase, the CPB phase where explantation of the native heart and/or MCS device and implantation of donor heart is performed, and the post CPB phase.

### Pre-CPB phase

The anesthetic management during this phase is largely dependent on the underlying diseases and conditions. In general, ACHD with advanced heart failure can be classified into 1) Patients with uncorrected defects, 2) Patients who underwent repair or palliation but developed heart failure, and 3) Patients with failing single ventricle physiology. Unfortunately, both national and international databases lack information on the underlying defects and prior palliative procedures of ACHD undergoing heart transplantation. According to case series, 40–50% of patients have single ventricle physiology with failing Fontan circulation [7,8]. Surgically or congenitally corrected transposition of the great arteries and systemic right ventricular failure is the next most common lesion. MCS has been increasingly used in ACHD with heart failure. It was < 4% of ACHD who were supported with MCS from 1993 to 1996, but the proportion rose to > 14% between 2010 to 2012 [5].

Fontan surgery underwent several modifications since its initial report in 1971 [10]. Currently, total cavopulmonary connection (TCPC), which directly diverts the superior vena cava (SVC) and the inferior vena cava (IVC) flow to the pulmonary artery (PA) without the right side of the pumping ventricle, is very common. This can be done by placing a conduit in the atrium (lateral tunnel Fontan) or placing a conduit outside the atrium (extra-cardiac Fontan) [11]. Often a fenestration is placed in the Fontan baffle to allow right to left shunt and have cardiac output in case pulmonary vascular resistance (PVR) is elevated. We still see a fair number of patients who had received atriopulmonary connection, which was the type of Fontan before TCPC. This is made by connecting the right side of the atrium to the PA. This circulatory repair fell out of favor due to the progressive dilation of the right sided atrium with subsequent low cardiac output and/or arrhythmias. They often present for cardioversion for arrhythmias and/or Fontan revision for TCPC. Due to the lack of right-side ventricle in the Fontan circulation mentioned above, systemic venous pressure needs to be elevated to drive blood to the pulmonary vasculature. Patients with Fontan circulation have increased venous stiffness and decreased splanchnic venous capacitance to accomplish this. The induction of general anesthesia can often cause vasodilation including splanchnic vasculature, which would significantly reduce preload in these patients. Volume administration is advocated prior to the induction of anesthesia for this purpose, but patients with failing Fontan are sensitive to volume loads. Using inotrope and vasoactive agents is indicated at the time of anesthesia induction to support their poor ventricular function as well as maintain vascular tone, as we have previously reviewed and published their anesthesia management [11]. Patients with failing Fontan could have elevated left atrial pressures due to poor ventricular function. As a result, they often have high Fontan baffle pressures. Fenestration in the baffle can allow right to left shunt to attenuate the elevation of baffle pressure. Often, they may develop venous-venous collateral to decompress elevated baffle pressures, which could contribute to further cyanosis. The presence of these collaterals should be delineated in cardiac catheterization for potential coil embolization if indicated. To compensate for cyanosis, aortopulmonary collaterals may develop in these patients. Failing Fontan patients may have a number of complications other than ventricular dysfunction. Arrhythmias, thromboembolism, protein losing enteropathy, and plastic bronchitis are among them. Protein losing enteropathy may be treated with steroids, and consideration for stress dose steroids should be given if there is significant hypotension resistant to vasoactive agents, though steroids should be given as part of the immunosuppressive regimens intraoperatively for heart transplantation. The presence of plastic bronchitis can complicate airway management. If its presence is suspected, bronchoscopy should be considered prior to endotracheal intubation.

Surgically corrected transplantation of the great arteries is seen in patients with dextro-transposition of the great arteries (d-TGA) who underwent atrial switch operation with Mustard or Senning. Atrial switch surgery was applied to patients with d-TGA before the era when arterial switch operation was established as the standard surgical procedure for the majority of these patients. Patients who underwent atrial switch operation have morphological right ventricle as the systemic ventricle, which might present with ventricular failure. In addition to systemic ventricular failure, any complications including any obstruction of atrial baffle with clot or anatomical narrowing need to be delineated prior to planned transplantation. Congenitally corrected TGA (l-TGA) also has morphological right ventricle as the systemic ventricle. Now these patients may undergo double switch operation consisting of atrial switch and arterial switch so that they have morphological left ventricle as a systemic ventricle. However, these patients often live life without any corrective procedures.

In addition, patients with MCS can present for heart transplantation. HeartMate II left ventricular assist device (LVAD) was first approved as LVAD for bridge to transplantation. HeartMate III LVAD and HeartWare HVAD are often used now. Thus, anesthesiologists who take care of them need to be familiar with the care of ventricular assist devices. It is important to know the physiology of each device. When only systemic ventricle is supported with the device, it is important to support the pulmonary ventricle or the pulmonary side of the circulation, which includes use of inhaled nitric oxide (NO) and intravenous milrinone therapy. We have previously reviewed anesthesia care for patients on MCS. Please refer to our previous publication for further detail [11].

As above, a number of ACHD who present to heart transplantation have had previous surgical interventions. It is critical to have good vascular access to be amendable for volume resuscitation in case significant bleeding is encountered. Often these patients have had a number of interventions done through the femoral arteries and veins for cardiac catheterizations and other procedures. Patency of these vessels for femoral access is an important piece of information to gather for potential femoral cannulation when difficult sternotomy is anticipated. If they are not available, alternative plan for cannulation for CPB should be well discussed with the surgical team.

### CPB phase/Post CPB phase

A number of ACHD patients have prior surgical interventions. Furthermore, the presence of aortopulmonary collaterals can often impede surgical field visibility. Dissection and explantation of heart and/or device can be difficult. Implantation usually consists of connecting SVC and IVC and left atrium cuff to the donor heart. However, reconstruction of vascular structures may be required. The procurement surgeon often has to obtain extended donor veins and aorta for this purpose. These complicated surgical issues may contribute to longer ischemic time. In the study by Karamlou, et al., the average ischemic time was 3.8 hours in the ACHD arm, while it was 2.9 hours in the non-ACHD arm [12].

As a part of the heart transplantation workup, the assessment of pulmonary vascular resistance (PVR) is mandatory. However, the measurement may not be accurate in ACHD due to 1) Presence of multiple sources of pulmonary blood supply due to the presence of aortopulmonary collaterals or shunt and 2) Low pulmonary blood flow [5]. In the United Network for Organ Sharing (UNOS) registry, 51% of non-ACHD had had a pulmonary wedge pressure > 20 mmHg, but the percentage of ACHD who had this high pulmonary wedge pressure was only 31% [5]. But ACHD had severe pulmonary hypertension more often than non-ACHD. With the combination of potential longer ischemic time and elevated PVR, donor right ventricular dysfunction should be anticipated in the post CPB phase. Intervention to reduce PVR including inhaled NO and intravenous milrinone should be considered. When a significant degree of aortopulmonary collaterals exist, left ventricle will be subjected to a significant volume load with high cardiac output. When the development of high cardiac output failure is suspected in the post CPB phase, earlier intervention such as coil embolization in the cardiac catheterization laboratory should be considered because identifying and ligating those collaterals intraoperatively is difficult. Other significant issues include bleeding control. Extensive dissection due to redo sternotomy, pre-existing coagulation abnormality or preoperative use of anti-coagulants contribute to this in addition to factors such as the effect from CPB run. These patients may also have preexisting renal and hepatic dysfunction in light of advanced heart failure. These could affect volume status, coagulation profiles, and drug clearance.

## Outcomes of Heart Transplantation in ACHD

Early postoperative mortality is higher for ACHD patients. In a systematic meta-analysis, the 30-day mortality was 17.4% in ACHD, while it was 7.4% in non-ACHD [4]. However, those who survived this earlier phase had better long-term outcomes. 10-year survival was 49.0% in ACHD and 40.7% in non-ACHD. The subgroup analysis of the 30-day mortality showed that the mortality of single ventricle patients who underwent palliation was significantly higher (43.8%) than that in non-CHD patients (14.4%) [4]. Primary graft failure due to longer ischemic times, stroke and hemorrhage are responsible for this difference. Appropriate precaution is needed for anesthesia care in this subset of patients. Interestingly, single ventricle patients without previous palliative surgery had similar 30-day mortality to non-CHD patients, but more data is needed to understand this outcome.

## Post-transplantation Consideration

Given that the long-term outcome of ACHD heart transplant recipient is better than non-CHD patients, it is easily expected that they could present for a variety of procedures requiring anesthesia care. Some of the procedures may be able to be done with sedation. ACHD often have extensive experience with previous hospitalizations and procedures. However, these experiences may not make sedation for seemingly simple procedures amendable for this patient population due to their anxiety. General anesthesia may be indicated in a number of cases.

A number of reviews and case reports have discussed the anesthetic considerations in heart transplant recipients for further review [13–17]. Cardiac deneravation occurs because the cardiac plexus is divided for transplantation. Donor atrium is responsible for generating the heart rate and rhythm. Although reinneravation has been described in transplanted heart, this is a very slow process and vagolytic drugs may have no or limited effect. The physiology of transplanted heart can be characterized by elevated filling pressures, increased end-diastolic and end-systolic volumes, low normal left ventricular ejection fraction, and a restrictive physiology [18]. Thus, stroke volume may be relatively fixed and their cardiac output may be dependent on heart rate. Thus, they are very sensitive to heart rate reduction. A unique issue around heart transplant recipients receiving general anesthesia is extreme bradycardia and subsequent cardiac arrest associated with the use of neuromuscular reversal agents. This has been reported with the use of neostigimine and sugammadex [13,19–22]. Because no and/or limited response to vagolytic drugs is anticipated, epinephrine should be immediately available. Regional anesthesia use for patients with a history of heart transplantation can be considered. This use has been successfully documented [23]. However, this should be reserved for patients who can tolerate the procedure given their potential anxiety.

## Conclusion

ACHD patients with heart failure increasingly undergo heart transplantation. They pose a significant challenge to anesthesiologists during heart transplantation and after transplantation, and understanding their pathophysiology would be critical for the further improvement of their outcomes.
